# Design principles of biologically fabricated avian nests

**DOI:** 10.1038/s41598-019-41245-7

**Published:** 2019-03-18

**Authors:** Hadass R. Jessel, Sagi Chen, Shmuel Osovski, Sol Efroni, Daniel Rittel, Ido Bachelet

**Affiliations:** 1 Augmanity Nano Ltd, Rehovot, 7670308 Israel; 2 Faculty of Mechanical Engineering, Technion, Haifa, 32000 Israel; 3 The Mina & Everard Goodman Faculty of Life Sciences, Bar-Ilan University, Ramat Gan, 52900 Israel

## Abstract

Materials and construction methods of nests vary between bird species and at present, very little is known about the relationships between architecture and function in these structures. This study combines computational and experimental techniques to study the structural biology of nests fabricated by the edible nest swiftlet *Aerodramus fuciphagus* on vertical rock walls using threaded saliva. Utilizing its own saliva as a construction material allows the swiftlets full control over the structural features at a very high resolution in a process similar to additive manufacturing. It was hypothesized that the mechanical properties would vary between the structural regions of the nest (i.e. anchoring to the wall, center of the cup, and rim) mainly by means of architecture to offer structural support and bear the natural loads of birds and eggs. We generated numerical models of swiftlet nests from μCT scans based on collected swiftlet nests, which we loaded with a force of birds and eggs. This was done in order to study and assess the stress distribution that characterizes the specific nest’s architecture, evaluate its strength and weak points if any, as well as to understand the rationale and benefits that underlie this natural structure. We show that macro- and micro-scale structural patterns are identical in all nests, suggesting that their construction is governed by specific design principles. The nests’ response to applied loads of birds and eggs in finite element simulations suggests a mechanical overdesign strategy, which ensures the stresses experienced by its components in any loading scenario are actively minimized to be significantly smaller than the tensile fracture strength of the nests’ material. These findings highlight mechanical overdesign as a biological strategy for resilient, single-material constructions designed to protect eggs and hatchlings.

## Introduction

Avian nests have a high degree of design variation across families which is translated to multiple functionalities. As they primarily serve as a location and apparatus for incubation of eggs^1–3^ and a safe place for offspring to develop^4^, nests’ are hypothesized to integrate parts with specific physical and mechanical properties, evolutionarily selected to provide comfort^5^, sexual signalling^6,7^, defense from parasites or pathogens^6,8^, and thermoregulation^9,10^. This suggests that construction is guided by specific fabrication programs whereas materials are deliberately selected for specific roles. However, deliberate selection of materials for specific functions in avian architectures has yet to be fully investigated^11^.

Birds that build nests by means of assembly, collect and join together materials to create a receptacle for the eggs. They use various construction methods that can be divided into piling up, molding, sticking together, interlocking, sewing, and weaving. The purpose of the various techniques is essentially to ensure that the nest remains attached to the nesting site and that it retains its physical integrity^12^. Some materials stick together by their inherent properties (e.g. mucus), while others require certain spatial relationships (e.g. branches)^12^.

Several studies have attempted to determine the factors that affect nest biomechanical characteristics by studying the construction materials and architecture. Common House Martins *Delichon urbicum* mold nests by placing large numbers of mud pellets to a growing nest rim. They enhance the mechanical behavior of the mud-based nesting materials, particularly in compression, with the addition of complex polysaccharide. The shape of the nest was shown to be an optimum structural system for the loads that are subjected by the bird and eggs^13^. In the nests of the Common Blackbird *Turdus merula*, the outer nest was composed of thicker, stronger and more rigid elements compared to the materials present within the structural wall and the cup lining. The outer nest components were more loosely arranged and are suggested to have a role in providing a supporting foundation framework for the nest^2^.

Other animals have also been shown to exhibit elaborate functions by principles of design and architecture, assembling materials in a non-random manner during the construction of nests and other structures, and their principles of design and architecture are of increasing interest^14^. Orangutans (*Pongo sp*.) build nests daily in trees by weaving branches together during construction for support and shelter on rest periods. The nests are built upon a solid base to which branches are woven together to form the base of the nest. They select stronger, more rigid materials for the outer rim of the nest compared to the weaker and more flexible materials used to construct the cup lining^5^. *Araneidae* orb-web spiders produce secretions from seven different glands, all of which are involved in aspects of web construction with different compositions and material properties^15^. The thread produced were shown to have high values of tensile strength and elasticity, allowing the web to absorb the sudden impact of even a large insect hitting the web without breaking^16–18^.

To date, mechanical and structural characteristics of nests built by assembling components or applying self-secreted materials are limited to only a few bird species^11,19^. Little is known about whether birds are generally selective of nest materials based on their biomechanical properties. In contrast to collected-materials builders, animals that build by the deposition of secreted endogenous material layers (i.e. additive manufacturing), such as bees^20^, silkworms^21^, spiders^22^ and swiftlets exhibit rigid, relatively consistent design principles, owing to their full control over the construction material deposition during the building process. This control enables some species to achieve mechanical diversity by modulating the biosynthesis and composition of the same material during construction^23,24^, a capability shared by contemporary human additive manufacturing technologies such as 3D printing^25^.

We focused on nests of the edible-nest swiftlet *Aerodramus fuciphagus*, one of a few avian species that use additive manufacturing to construct its nest, composed entirely of saliva^26^. The male swiftlet fabricates the nest by manipulating threads of saliva on the nearly vertical surfaces of caves^26,27^, taking approximately 35 days to complete a single nest^28^. The nest begins as a large pad of saliva spread over the substrate. A lip is then added to it which gradually increases in size until a spherical half-cup shape is formed^26^. Utilizing its own saliva as construction material allows the swiftlets full control over the structural features at a very high resolution in a process similar to extrusion-based 3D printing (such as fused filament fabrication), an additive manufacturing technology^29^. The dried saliva contains mostly glycoproteins^30^, but how this composition is modulated in different nest parts is unknown.

This study combines computational and experimental techniques to study the structural biology of nests fabricated by the edible nest swiftlet. Specifically, we examined how material properties integrate with structural design, and the mechanical properties of different nest parts. We aimed at identifying weak points in the structure of the swiftlet nest, and elucidating the biological-mechanical rationale behind its design. This was done by creating high resolution finite element (FE) models of swiftlet nests, generated by segmenting μCT scans of purchased nests. Defining material properties for FE analysis was done by *in-situ* uniaxial tensile testing of the nests material, since the material properties of the salivary secretions have been unknown to date. Finally, these models were loaded with the force of birds and eggs and were used to study the behaviour of this natural structure. These FE models were used to predict how the nests respond to these prescribed loads and displacements.

## Results

**Figure 1:**
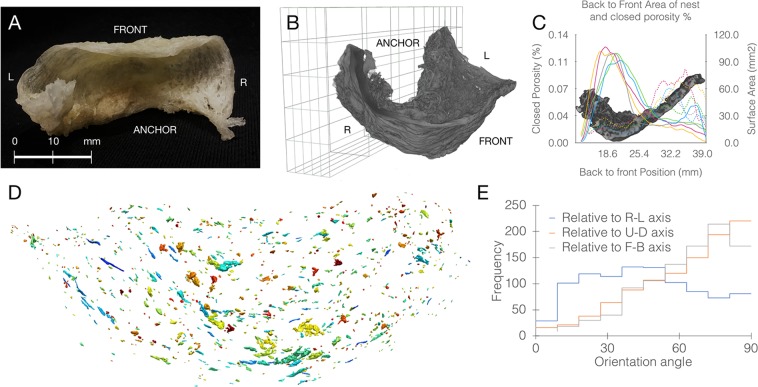
3D characterization of the edible swiftlet nest. (**A**) A representative nest photograph showing the overall structure with orientation, bar = 20 mm. (**B**) 3D model of the nest, reconstructed from the μCT scan, oriented relative to the wall to which it is anchored. (**C**) Similarity between nests at the microstructure level. The distribution of two representative properties, surface area and percentage of closed pores revealed by the μCT scan, across the nest in the back-to-front direction. A collection of n = 5 nests showing that they exhibit the same pattern. Continuous lines are surface area and dotted lines are percentage of internal closed porosity. (**D**) Multi label pore mask of closed pores in the nest. (**E**) Quantitative analysis of pore orientation, demonstrating that the pores are approximately orthogonal to the up-down (U-D) and front-back (F-B) axes, and are aligned with the right-left (R-L) axis, as expected of a horizontally-biased structure. D and E were performed on one of the nests.

Collected swiftlet nests (Fig. 1A, Supplementary Note 1) were nearly identical in their overall shape, dimensions (77.5 × 39.8 mm ± less than 10% to each dimension), and weight (5.93 ± 0.61 g), which was also identical to measurements reported in older studies^26,31^. We obtained complete structural 3D information of the nests by X-ray microtomography (µCT) set to a resolution of 34 μm (Fig. 1B). This scan revealed the distribution of material density, porosity and construction pattern across the different parts of the nest. Interestingly, all nests were shown to exhibit the same distribution and construction pattern, with nest walls being made up of thin threads (approximately 0.25 mm thick) while the anchoring was formed from thicker, more dense, thus stronger material. A section view reveals a highly repetitive, geometrically graded structure with a high surface area in the anchoring. The surface area gradually declines towards the ends (Fig. 1C). The segmented µCT scan revealed empty spaces fully surrounded by material on all sides (small closed pores). These pores in between the strands of solidified saliva showing low porosity near the base, becoming higher towards the ends. The multi-label pore mask revealed that the strands of the solidified saliva form a horizontally-biased structure with pores perpendicular to load direction (Fig. 1D,E). These findings suggest that swiftlets carry out a precise fabrication program, integrating simultaneous control over structural pattern and construction material properties.

**Figure 2:**
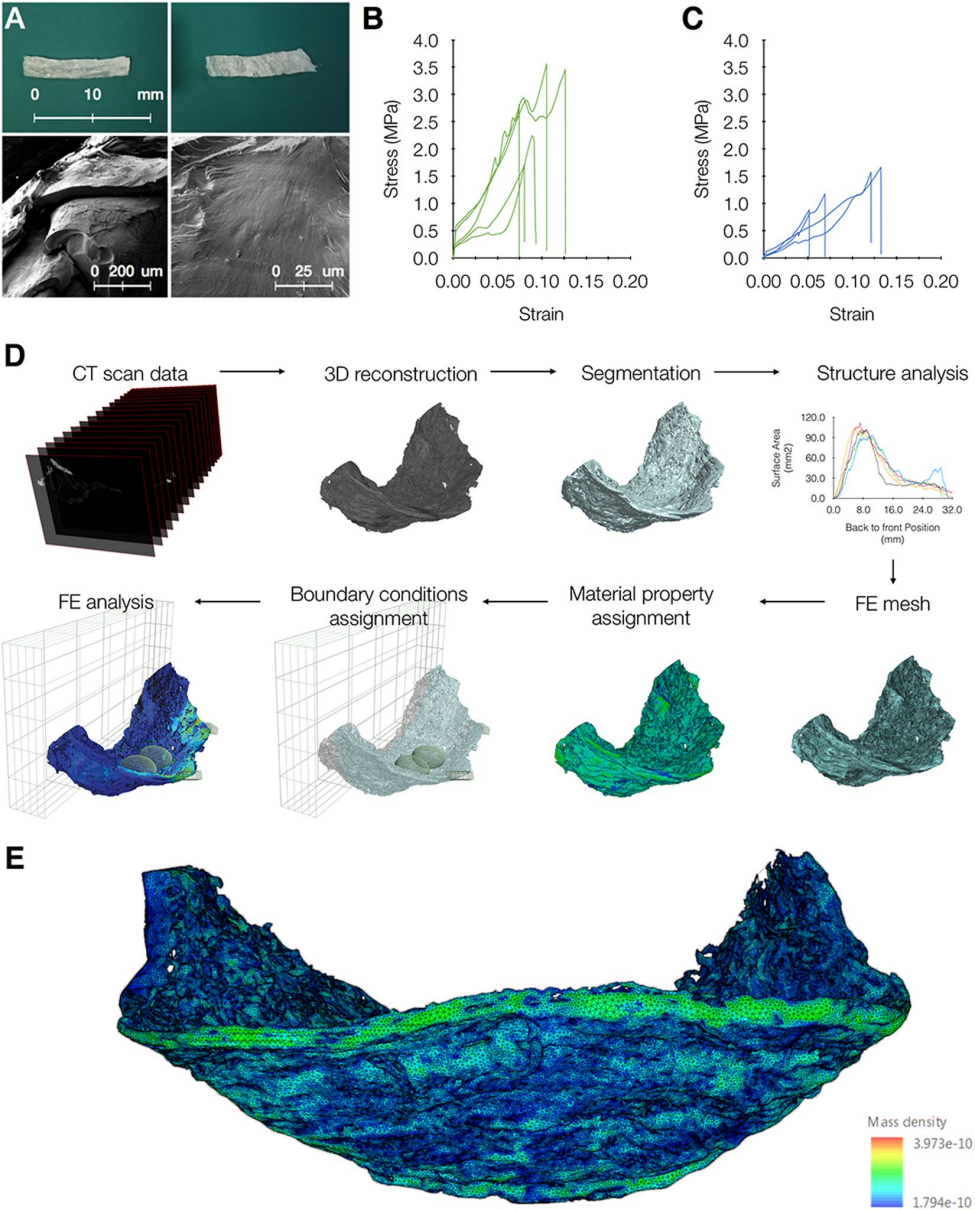
Mechanical characterization of nest material. (**A**) Samples of nest slices used for tensile testing. Left, slice cut along the fiber direction; right, slice cut perpendicular to the fiber direction. Bottom panel shows SEM image of fracture under tension. Fracture surfaces of both specimen types showing typical tensile fracture characteristics from representative tensile tests. Different fibres in the same nest were observed to fail at different locations, and have different characteristics. While some fibres were completely flat, with no observable features on the fracture surface, others were rougher and exhibited clear markings indicating the fiber’s crack growth direction. (**B**,**C**) Altogether, these tests yielded a mean breaking stress (peak stress in the stress-strain curve) of 2.75 ± 0.79 MPa and a mean elastic modulus of 155 MPa. (**D**) Workflow for nest reconstruction, from computer tomography (CT) scans to the finite element (FE) model: μCT scanned image files imported into ScanIP. Background image data segmented and analyzed within scanIP. +FE module within ScanIP is used for defining contact entities and generate the FE mesh. FE mesh imported into Abaqus for assignment of material properties and boundary conditions. Finally showing +FE Free Mesh after analysis in Abaqus. (**E**), A high- resolution finite-element (FE) model of the nest created based on the 3D model and the mechanical measurements, with material density represented by color scale (blue, least dense; green, most dense). Shown is the FE model of one of the studied nests.

The orientation, distribution, and magnitude of strains and stresses within a structure depend upon the applied load, but also on material properties and structural organization. However, the material properties of the swiftlet nests were unknown. Therefore, we measured these properties by *in-situ* uniaxial tensile testing under scanning electron microscopy. In order to obtain well-defined specimen shapes, rectangular slices were cut from different nest regions and in different directions (Fig. 2A). From the results of these tests, nest parts were clustered into two types based on tensile behavior and their microstructure. In ‘Weak’ slices, the majority of fibers were oriented 45–90 degrees with respect to the loading direction. The failure process was observed to be composed of both fracture of fibers as well as interlayer breakage, followed by an immediate stress drop and catastrophic fracture. In ‘Tough’ slices the fibers were aligned with the tensile load. The stress-strain behavior exhibited a nonlinear pattern, with several stress drops associated with the failure of individual fibers prior to ultimate failure. (Fig. 2B,C). To clarify, this experiment was based on nest slices cut in different directions (see Supplementary Note 2). We show that when the slice is cut along the fiber direction (longitudinal) the slices are mechanically stronger than those cut in transverse. To best show this, we aggregated readings received from several replicate slices on the same graphic system. The overall ductility (strain to fracture) of the fiber mat structure is limited (ca. 0.125) irrespective of the fibers’ orientation. Yet, those results represent more of a structural than a material response due to the complex mat’s architecture. These results yielded a mean fracture stress, defined as the peak stress, of 2.75 MPa with a standard deviation of 0.79 MPa and a mean elastic modulus of 155 MPa.

High element quality FE meshes showing microstructure features were then generated, based on these material measurements, in order to calculate the stresses and strains resulting from applied loads (Fig. 2D,E). Each bird weighed 16 gr, noting that this is approximately twice the weight of a typical nest. All loads were modeled as external loads applied onto the nest in the vertical axis parallel to the direction of gravity, at specific positions where eggs and birds are found, in the nest and on the nest rim, respectively. The main loading scenario, which represents the worst case loading scenario, involves two adult swiftlet birds and two eggs, as documented for this species^32^.

**Figure 3:**
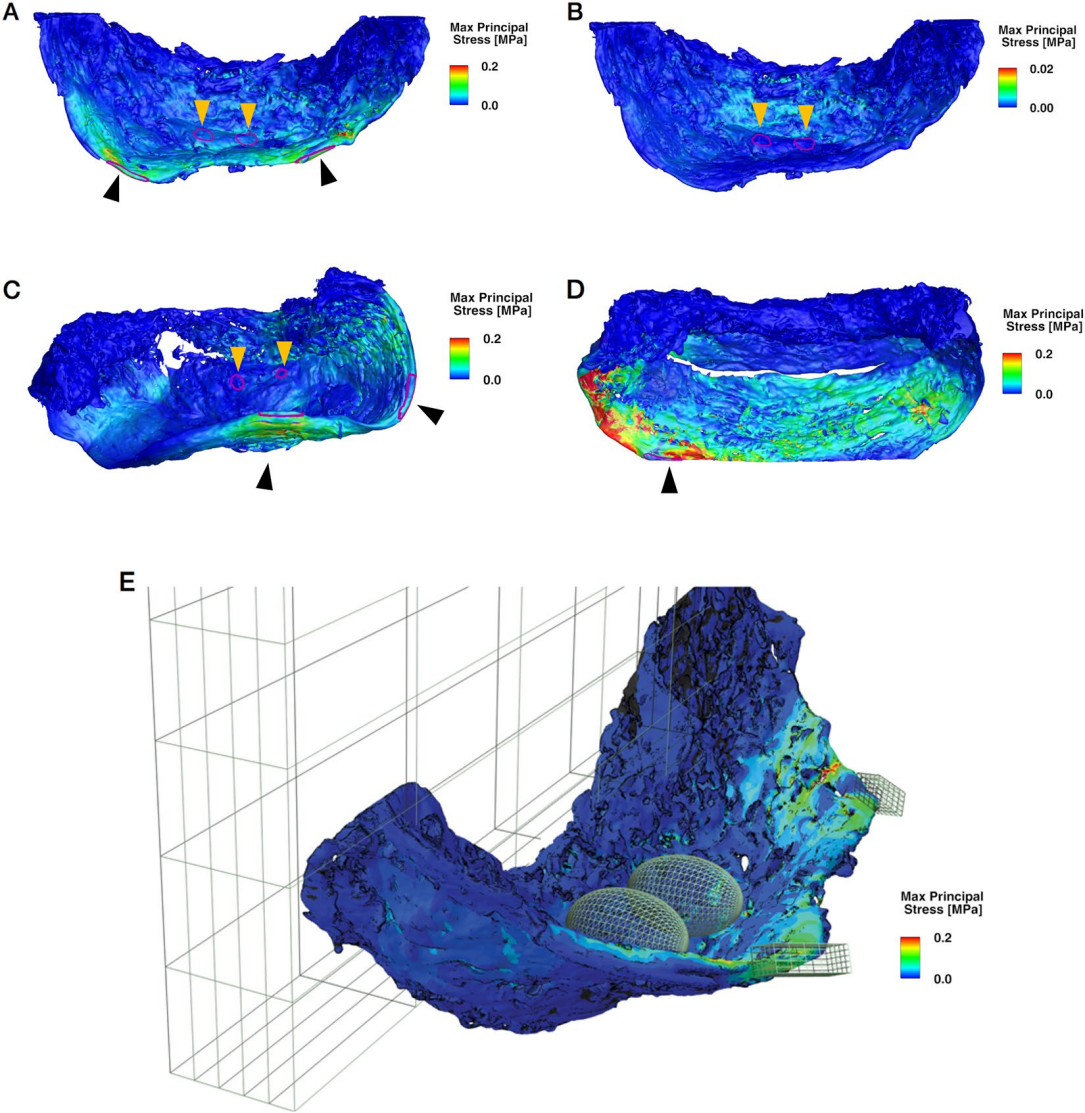
Finite element simulations results showing the maximum principal stress at the end of each linear static loading scenario. (**A**,**C**,**E**) Showing worst case loading scenarios, namely two eggs and two birds positioned on the nest rim to apply a maximum bending moment on the nest’s fixation to the wall. (**A**–**D**) Showing top view of loading scenarios. (**A**) Worst case loading scenario with two adult birds and two eggs. Black arrowheads indicating bird standing positions on nest rim. (**B**) Loading scenario with two eggs and no birds. Orange arrowheads indicating egg positions. It is clear that the “egg-region” is experiencing lower values of stress. (**C**,**D**) Effect of damaged sites in the structure on the response to loads, showing that the stress was localized along fault lines that originate at those sites. Arrowhead indicates position of loading. Panel D shows that the anchor region is mostly isolated from the stresses induced by loading from the positions indicated by black arrowheads. (**E**) The FE model result of maximum principal stress of the nest under a complete loading scenario. ROIs’ are shown to highlight the regions where loading is applied. Birds are represented by cuboid ROIs on the nest rim, eggs as ellipsoid ROIs and anchoring area is presented within the grid box.

Throughout this section, we will consider the maximum principal stress (referred to as “stress” in the sequel), as the latter is a well-accepted fracture criterion for brittle materials, a category to which the nest’s material belongs to as a first approximation. In all cases, the resulting stress was distributed along the fiber direction and towards the wall anchoring (Fig. 3A,C). Analysis of the stress in the model revealed it reaches its maximum value where the birds stand (black arrowheads) as shown in Fig. 3, with a peak point value of 0.56 MPa. The numerical simulations revealed that the stress at all locations is significantly smaller than the fracture strength of the bird nest material. The most highly stressed section is the outer rim of the nest, where the birds stand. Theoretical fracture of the rim may lead to a brittle failure which won’t endanger the nest itself, and more precisely the eggs since it results from a propagation and connection of elongated and flattened ellipsoid pores along the rim, horizontally. Moreover, the nest’s section where the eggs are positioned was essentially stress-free in all scenarios, effectively insulated from stresses imposed by the adult birds themselves due to the fibers conducting and geometrically distributing the stresses in the horizontal direction (Fig. 3B).

According to the simulation results, stress contours showed that the applied loads and the resulting stresses were distributed over the unbounded rim section of the nest along the fiber direction. The spherical half-cup shape of the nest acts to reduce the stress magnitude and leads to a relatively homogeneous and low stress distribution in the anchoring to the wall. The anchor region remained nearly completely isolated and experienced very little or none of the stresses induced by the applied loads (Fig. 3D,E).

Stress decays significantly (with or without eggs) from the nest’s rim towards its anchoring. This is the *combined result* of the *nest’s geometry*, a half-cup, that is characterized by a graded increasing cross-sectional area, and the fact that the material is denser, thus stronger in this location.

## Discussion

Edible-nest swiftlets construct nests entirely out of saliva by manipulating threads of saliva on nearly vertical surfaces^26,27^. Our analysis of the structural and mechanical properties of the edible swiftlets nest showed that the base of the nest was composed of significantly thicker material than the walls and the outer rim, which in turn had significantly more closed pores. 3D reconstruction of µCT scans showed elongated closed pores in between the strands of solidified saliva that were formed due to the horizontally biased fabrication method. A remarkable feature of the studied nests was the similarity in their macroscopic (weight, shape) and microscopic (pore area and distribution) properties, highly suggesting that the nests are constructed according to the same specific design principles.

The finite element analysis of nest mesh-models revealed that when applying forces of two birds on the nest rim, the resulting stresses were highest at the outer rim of the nest, where the birds stand, and were distributed along the fibers direction towards the wall anchoring. Moreover, the nest’s section where the eggs are positioned was essentially stress-free, effectively insulated from stresses imposed by the adult birds themselves due to the fibers conducting and geometrically distributing the stresses in the horizontal direction. Our results indicate that the nests were fabricated with specific physical and mechanical properties for the purpose of holding and supporting two birds and two eggs.

The shallow half-cup shape of the swiftlet nests is common in other bird species as well^3^. Similarly fashioned nests of the Common House Martins are also constructed on vertical surfaces, however unlike swiftlets they employ the roof as additional support for construction. The elongation of the swiftlet nest could help to bear the structure and effectively distribute the loads without this additional support. This could be investigated *in-silico* by digital manipulation of nests’ FE models’ into new shapes. Such studies would provide further insight into the selection of nest architecture in relation to prescribed loads and nest location.

The relative cost-effectiveness of self-secreted building materials in comparison to collected material is difficult to calculate and is likely to vary between species^33^. The swifts (*Apodidae*), a family containing about 90 species^34^, are the only bird family which uses saliva as a construction material. While the edible-nest swiftlet is the only species to compose the nest entirely out of saliva, other species such as black nest swiftlets *(A. maximus)* integrate collected materials such as the birds’ feathers together with the saliva^35^. Evidence indicates that secreted materials have evolved to fulfil the specialized functions of bearing loads in tension, whereas collected materials have continued to fulfil the less specialized task of bearing loads in compression^33^. A phylogenetic study examining the evolution of nests built from saliva and from collected materials in the swifts would be useful and informative. It would be interesting to study whether the choice of integrating collected material affects the mechanical properties of the nest structure or may be of significance as insulation materials^36^ only.

The distribution of material within the studied nests was not random as in other nests studied^2,3,12,37^. Rather, the base, originally glued to the wall, was constructed with a significantly higher material surface area compared to the rim of the nest. This property gradually decreases from the base to the rim, as analysed using the μCT scan slices in the back-to-front direction. The spreading of material over the wall surface may well reflect a load bearing role of this architectural region. This is precisely the locus where the bending stresses are expected to be maximal when the nest is loaded with birds and eggs. However, the wider anchoring area reduces the local stresses, due to its higher moment of inertia, thereby preventing nest detachment from the wall.

The distribution of closed pores within the nests studied was similar in all nests. The rim, where birds stand, and nest wall showed significantly higher internal pore count compare to the area the eggs are laid and the anchoring to the wall. The internal pores in between the saliva fibres are generated by a horizontal deposition of material. Analysis of pore direction and distribution revealed that a theoretical fracture of the rim may lead to a brittle failure which won’t endanger the nest itself, and more precisely the eggs, since it results from a propagation and connection of elongated and flattened ellipsoid pores along the rim, only in the horizontal direction.

The design of the nest appears to be optimized in a way that the relatively thin wall located between the eggs and the rim, successfully copes with the stresses imposed on it by both eggs and the birds. This is achieved by two design principles: a) a gradually decreasing cross section towards the outer rim; and b) a horizontally biased fabrication strategy, with fibers spreading the stresses in the horizontal direction. Due to these principles, the stresses experienced by the various components of the nest are significantly smaller than the nest’s material fracture strength as we measured. The cross-section structure together with fiber orientation act to spread and minimize the stress on the critical part of the nest, where the eggs or hatchlings are laid. The peak stresses that result from this minimization are then supported by a “smartly” optimized structure, whose design is redundant in order to successfully bear stress levels that will not cause fracture of the nest. This has also been suggested for nests in other species^13^.

Further research into engineering by animals could yield valuable insights concerning the integration of design and materials to accomplish a wide range of objectives and function in various environments. Our study shows how a single material, distributed properly across a specific structure, could be used for constructing a sustainable and resilient structure. The design principles of a structure such as the edible swiftlet nest, which dictate the relationship between structure and material usage, could yield fascinating insights into the study of animal made structures, mainly ones made by organisms that are capable of building complex structures using only local or self-produced materials^38–41^.

## Methods

### Nests

Five nests were purchased from commercial bird nest farms in Selangor, Malaysia. The nests were harvested from the farms. They were shipped directly to our laboratory. On receipt of the nests, they were immediately scanned. The vendor confirmed that the provided nests were cleaned and processed without bleaching agents, and untreated with coloring or artificial preservatives. The nests were stored in separate closed containers with a relative humidity of 80% and a temperature of 25 °C throughout the research (Fig. S1).

### CT scans

μCT scans were performed on a SkyScan 1176 high-resolution μCT (SkyScan, Aartselaar, Belgium). After adjusting the appropriate parameters for scanning, each nest was positioned on the specimen stage and scanned with an isotropic resolution of 34.04μm, rotational step of 0.700 degrees, and a 41 ms exposure time (tube voltage 40 kV, tube current 600 μA with no filter).

### Image processing

μCT datasets were imported into Simpleware ScanIP M-2017.06-SP1 (Synopsys, Mountain View, USA), an image processing software used to visualize and segment regions of interest (ROIs) from volumetric 3D data. This software imports a stack of images from μCT slices in a wide variety of software formats (in this case, approximately 2500 bitmap files), allowing steps of visualization and assisted segmentation based on image density thresholding of different grayscale intensities. The following tools and workflow described were applied to all five nests. Image sequence of each μCT scanned nest was imported with a pixel spacing value of 34.04um in x,y and z with a background type of 8-bit unsigned integer. The image sequence was resampled by linear interpolating to a pixel size resolution of 68.1μm to downsample image data. The segmentation was then used to generate the volumes (binary volumes) that are called masks, which define how the objects fill the space. Several segmentation tools were used to create the masks from the background image data, which were modified until they showed a satisfactory mask. There are some artifacts and noise from μCT data, which can be corrected by filtering when the images are reconstructed. A threshold tool was used for segmenting the nest models based on grayscale intensities. Changing the threshold values of two-dimensional regions on the imported stack of images was done in order to select only the nest and exclude background noise. Grayscale boundaries were set to a lower value of 40 and an upper value of 255. A Flood-fill tool was used to remove non-connected artifacts from the mask, this algorithm is a connectivity-based algorithm and was used on the active mask. A recursive Gaussian filter was used with a sigma value of 1 in each direction to reduce image noise and reduce detail level. Closed pores with less than 125 voxels were selected and added to the mask to reduce computational time and to create a higher quality of the generated elements. ScanIP was used for the 3D volumetric visualization, analysis, and measurement. The morphometric parameters of the whole nest were calculated by the software. The following parameters used were: mass density, the volume of the nest, nest surface area, and pore analysis. Average mass density was defined as the ratio of nest mass (measured with a scale) to nest volume measured by scanIP 3D mask analysis. Measuring the distribution of the mass density, surface area and closed pores along different axes of the nest was done by generating a segmented mask for the nest and a separate segmented mask for the closed pores. A Slice-by-Slice script was written for slicing the masks in yz, xz and xy coordinate planes and finally the data of the pore and the nest masks were analyzed in every slice. A pore multi-label mask was generated from the segmented mask of the closed pores. Generating the multi-label mask was done to interactively visualize and analyze the pore mask that contained several regions (scattered pores in between the strands of saliva). The pore multi-label mask was created by labeling disconnected regions within the pore mask, where each distinct region was given a separate color.

### FE generation

**+**FE mesh generation module of ScanIP (v.M-2017.06) was used for conversion of the segmented 3D image data into high quality volumetric meshes. The +FE Free mesh creation algorithm was used to ensuring models whose geometric accuracy is high capturing the highly detailed nest microstructure with a true representation of porosity in the structure. The resultant smoothed, all tetrahedral FE meshes contained approximately 5M elements with a mean edge length aspect ratio of 4–5 and a mean in-out aspect ratio of 0.8–1. Defining contact entities and node sets was performed in ScanIP. Once the mesh was generated, an input (Abaqus volume) file was exported for the FE analysis. In the exported mesh, the node sets were selectable in the solver for applying boundary conditions/loads.

### Tensile testing

The brittle nest sample was softened before cutting by suspending them in distilled water for 20 minutes at room temperature (25 °C). After suspension rectangular pieces were cut using a scalpel at three different directions - 0° (longitudinal), 45° and 90° (transverse) angle to the hardened saliva fiber direction (Fig. S1). Next, the samples were flattened with magnets from both sides and dried in a desiccator for 4 days. A dedicated sample holder was printed in a Stratasys Connex3 3D printer using transparent VeroClear material with a glossy finish and SUP706 support, which was removed in an alkaline cleaning solution (Fig. S3). Each rectangular specimen was measured and attached to a sample holder using epoxy glue. The gauge length was measured after the glue hardened. All specimens were measured in several locations distributed along the specimen to determine their mean cross section area (A_0_). A quasi-static uniaxial tension test was carried out using a Kammrath &Weiss loading stage equipped with a 100N load cell (accuracy of 10^−3^N) to probe the mechanical behavior of the nest specimens. We stretched the specimens using a tensile module with a symmetrical cross head velocity of 1.3μm per second measuring the force (F) required to cause a given extension (ΔL). Force-displacement (F-ΔL) plots were converted into engineering stress-strain (σ−ε) curves by dividing force (F) by the initial cross-sectional area (A_0_) and displacement (ΔL = L−L_0_) by the initial length (L_0_)

$$\sigma =\frac{F}{{A}_{0}}\,{\rm{and}}\,\varepsilon =\frac{{\rm{\Delta }}L}{{L}_{0}}$$

Mechanical parameters like elastic modulus (*E* = σ/ε) and maximum tensile strength were directly obtained from the stress strain curve. The ratio of stress to strain, given as elastic modulus and derived from the linear regime of the curve, is a measure of the specimens stiffness^42^.

### Scanning electron microscopy (SEM)

Fractured surfaces of nest samples were mounted on aluminum stubs and coated with gold using a Bio-Rad E5000 Sputter Coater. Images of specimens after fracture were then acquired on a Mira3 (Tescan) scanning electron microscope operated at 10 kV in high vacuum mode. The tensile experiments were recorded at 0.5 kV using a beam deceleration voltage of 5 kV.

### FE analysis

FE meshed models were imported for use into the Abaqus/standard 6.14 package^43^. The material properties were taken directly from the tensile test data, using properties obtained from averaging the horizontal specimens, due to the observed horizontal bias and the orthogonal directions of forces applied by weights. The input data was set up following the nominal stress-strain curve. Due to the small deformations, a linear elastic model was adopted despite the nonlinear behavior observed at larger strains (Elastic modulus = 155 MPa, Poisson’s ratio = 0.3), the latter being most-likely due to geometrical nonlinearity rather than material. This assumption was verified by ensuring that the maximum stresses developed in the FE model did not exceed the stress at which the experimental curve deviates from linearity. The material was assumed to be isotropic (at the fiber level) where the structural anisotropy is arising due to the geometrical arrangement of the fibers as captured from the μCT scans. Body forces (gravity) were included in the simulation following the relative density taken from the μCT scan data. The loading scenario reported here, assumed a worst case loading condition with two adult swiftlet birds and two eggs, all of which are modeled as external loads applied on the nest in the gravity direction. For each nest an additional simulation was performed with a loading of two eggs solely to study their loading effect relative to the loading of the birds. The loading areas predefined in ScanIP contains a certain number of nodes, on which each of the external loads is homogeneously distributed. The node sets include two defined areas on the rim of the nest where the force of a bird is applied and two areas where the force of an egg is applied in the center of the nest. The forces applied by each bird and each egg are 16 g = 0.1569 N and 1.2 g = 0.0117 N, respectively^28,32^. For each nest FE model, the node sets in the geometrical locations at which the nest is in contact with the wall, were constrained to be fully pinned (i.e. zero displacements in all directions). Mathematical equations help predict the behavior of each element and then adds up all the individual behaviors to predict the behavior of the actual nest object. Stress and strain distributions in each nest have been calculated.

## Supplementary information

Supplementary info
