# Supplementary material for: Design principles of biologically fabricated avian nests
Source: Sci Rep. 2019 Mar 18;9:4792. doi: 10.1038/s41598-019-41245-7 (PMC6423035; doi:10.1038/s41598-019-41245-7)
Supplement: Supplementary file 1 — Supplementary info [file 41598_2019_41245_MOESM1_ESM.pdf]

Design principles of biologically fabricated avian nests

Hadass R. Jessel[1,3], Sagi Chen[2], Shmuel Osovski[2], Sol Efroni[3], Daniel Rittel[2], Ido Bachelet[1,4]

1. Augmanity Nano Ltd, Rehovot, 7670308, Israel,
2. Faculty of Mechanical Engineering, Technion, Haifa 32000, Israel
3. The Mina & Everard Goodman Faculty of Life Sciences, Bar-Ilan University, Ramat Gan 52900, Israel
4. Address for correspondence: 8 Hamada Street, Rehovot 7670308, Israel. E-mail address: dogbach@gmail.com.

Supplementary Notes

Supplementary note 1: Nests

Supplementary Snote 2: Tensile testing

Supplementary note 1: Nests

Nests were purchased from commercial bird nest farms in Selangor, Malaysia. Only nests cleaned and processed without bleaching agents, and untreated with coloring or artificial preservative were purchased. The nests were stored in separate closed containers at constant humidity and temperature throughout the research.

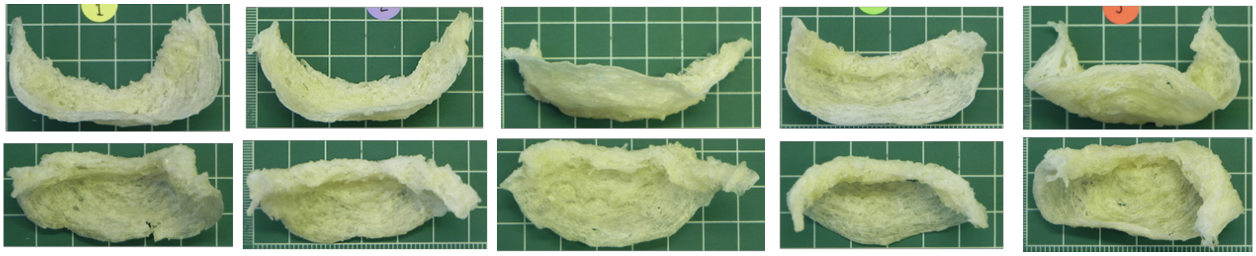

Figure S1. Photographs of 5 representative nests on centimeter grid.

Supplementary note 2:

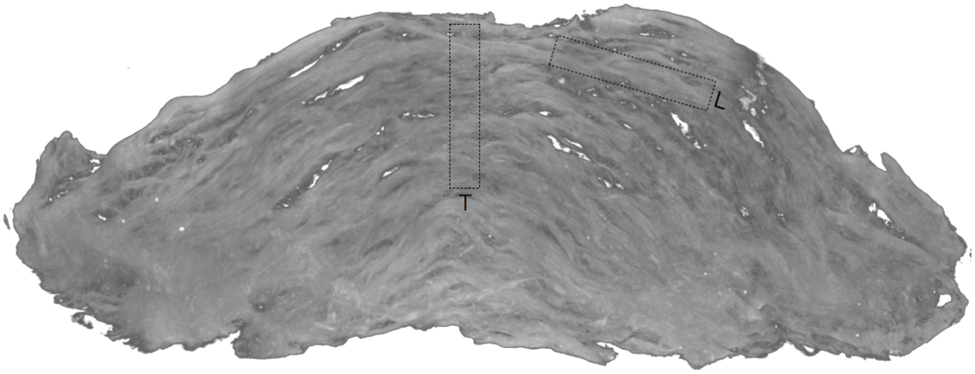

Figure S2. Slice cutting directions, used in tensile testing.

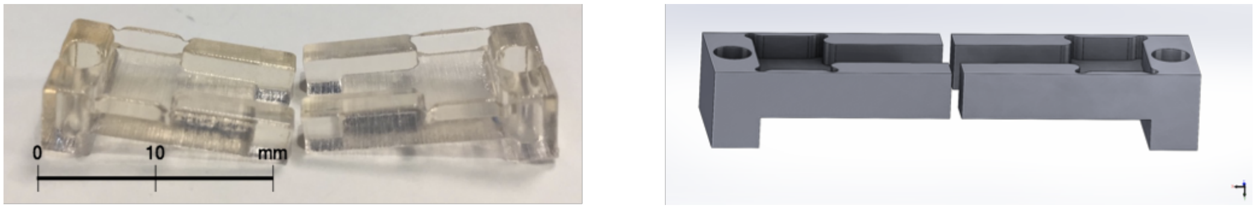

Figure S3. Plastic holder design (right) and printed holder (left).

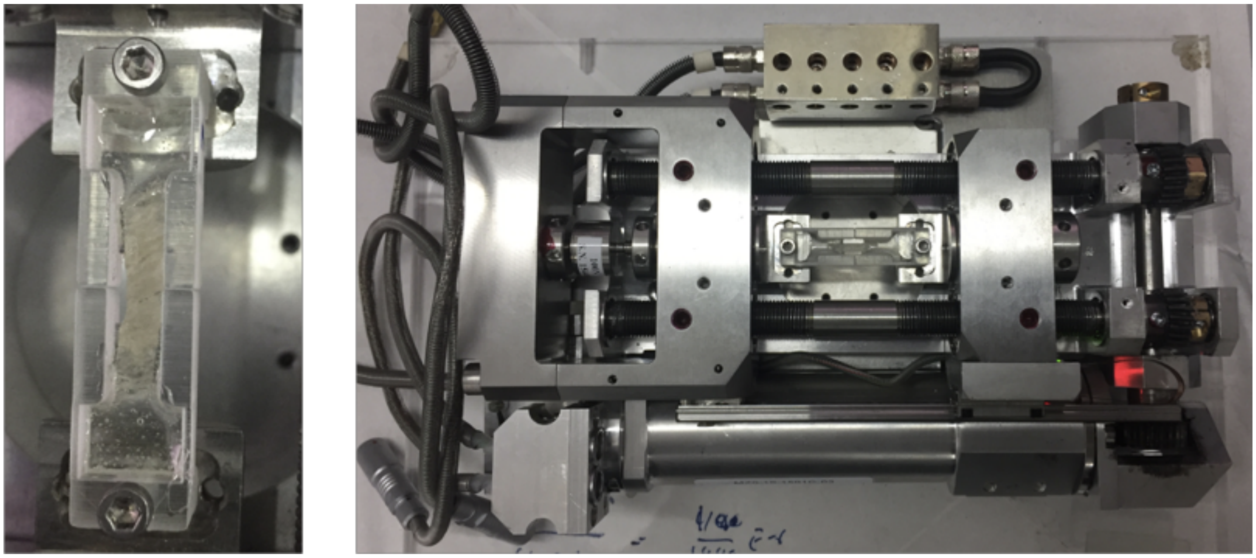

Figure S4. Tensile test in action. Left, nest slice within the sample holder. Right, holder within the tensile stage.
